# Failure Modes in Orthopedic Oncologic Reconstructive Surgery: A Review of Imaging Findings and Failure Rates

**DOI:** 10.3390/curroncol31100465

**Published:** 2024-10-17

**Authors:** Anuj Shah, Fabiano N. Cardoso, Felipe Souza, Julien Montreuil, Juan Pretell-Mazzini, H. Thomas Temple, Francis Hornicek, Brooke Crawford, Ty K. Subhawong

**Affiliations:** 1 Miller School of Medicine, University of Miami, Miami, FL 33136, USA; anujshah@med.miami.edu; 2 Department of Radiology, Miller School of Medicine, University of Miami, Jackson Memorial Hospital, Miami, FL 33136, USA; 3 Department of Orthopedic Surgery, Miller School of Medicine, University of Miami, Miami, FL 33136, USA; 4 Miami Cancer Institute, Division of Orthopedic Oncology, Baptist Health System South Florida, Plantation, FL 33324, USA

**Keywords:** orthopedic oncology, reconstructive surgery, failure modes, Henderson classification, revision surgery, limb salvage, endoprosthesis, allograft

## Abstract

Limb salvage surgeries utilizing endoprostheses and allografts are performed for a variety of oncologic conditions. These reconstructions can fail and require revision for many reasons, which are outlined and classified into mechanical failures (soft tissue failures, aseptic loosening, structural failure), non-mechanical failures (infection, tumor progression), and pediatric failures (physeal arrest, growth dysplasia). Distinct radiologic and clinical findings define specific failure subtypes but are sparsely illustrated in the radiology literature. Specifically, an understanding of the organizational structure of the failure modes can direct radiologists’ search for post-reconstruction complications, enhance an appreciation of their prognostic significance, and facilitate research by standardizing the language and conceptual framework around outcomes. The purpose of this review is to highlight the key radiologic findings and imaging studies of each failure mode in orthopedic oncologic reconstructive surgery in the context of risk factors, failure rates, prognosis and survival statistics, and clinical decision-making regarding chemotherapy, radiation, and revision surgery.

## 1. Introduction

Limb salvage surgeries can be performed for a variety of neoplastic conditions and utilize many reconstructive techniques, including endoprostheses, autografts, allografts, and allograft–prosthetic composites (APCs). Bulk allografts are often utilized for large bone defects where they enable the incorporation of the host–allograft junction, restore bone stock, and facilitate soft tissue reconstruction [1,2,3]. Biologic reconstructions also allow for improved load bearing, distribution, and more physiological range of motion while avoiding the mechanical failures that may be encountered with endoprostheses. However, allografts are vulnerable to complications, including graft resorption, nonunion, fracture, and infection [4]. Endoprostheses are widely used in orthopedic oncology for significant periarticular and metaphyseal defects after tumor resection as well as in the context of failed allografts, but they are similarly at risk for complications such as soft tissue and hardware failures as well as infection [1]. Tumor recurrence remains a risk no matter the reconstruction type.

The endoprosthetic reconstruction failure modes were outlined by Henderson et al. in 2011 and soon expanded to include both prosthetic implants and allograft constructs in 2014 [5,6]. This classification groups the failures of limb-preservation surgery into three categories—mechanical, non-mechanical, and pediatric failures—with six failure modes in total and further subcategorizations for each mode (Table 1 and Table 2). Although certain failure modes such as aseptic loosening and infection are more prevalent than others, developing a more thorough understanding of each failure mode is crucial to a systematic understanding of their frequencies and the associated patient morbidity and mortality [7,8].

The purpose of this review is to highlight key radiologic findings and imaging studies of each failure mode in orthopedic oncologic reconstructive surgery in the context of the risk factors, failure rates, prognosis, and indications for revision surgery.

## 2. Failure Modes in Orthopedic Oncologic Reconstructive Surgery

### 2.1. Type 1 Failures—Soft Tissues

Type 1 failures after endoprosthetic and biological reconstruction are soft tissue failures [5,6].

**Figure 1:**
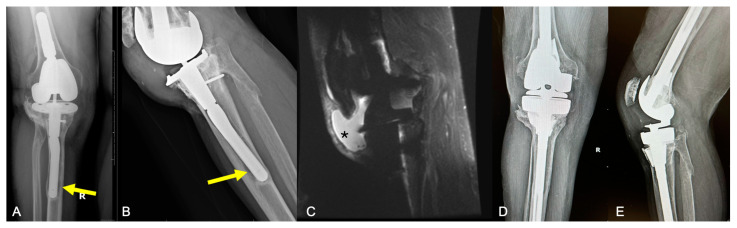
A 67-year-old male with a remote history of complex endoprosthetic reconstruction after resection of giant cell tumor of bone of the proximal tibia, now presenting 40 years after the initial reconstruction with knee pain and inability to extend the knee after kneeling. (**A**) AP and (**B**) lateral radiographs show a hinged knee arthroplasty with patella alta and prepatellar soft tissue swelling suggestive of patellar tendon tear. Also present is a concomitant aseptic loosening of the tibial component (yellow arrows) with posterior stem migration in relation to the initial soft tissue failure. (**C**) Sagittal fat-suppressed T2-weighted MRI confirms a full-thickness patellar tendon tear, with a large infrapatellar defect (*) that allowed wear-induced synovitis and joint effusion to decompress into the pretibial soft tissues, constituting a type 1A failure. Other surrounding ligaments show poor scar remodeling. The patient underwent revision total knee arthroplasty with extensor mechanism reconstruction using the Mayo Clinic Marlex Mesh [21], with (**D**) AP and (**E**) lateral radiographs taken 4 months post-operatively demonstrating secure femoral and tibial components.

Limb salvage surgery after an oncologic resection should involve the careful preservation of tissues to maximize function when possible. The use of soft tissue or muscle flaps is sometimes needed to provide adequate soft tissue coverage and optimize function and stability [9]. When this process fails or is performed inadequately, type 1A failures can occur, which involve the limited function of the prosthesis and limb due to insufficient muscle and ligament attachment. This limited function can be seen through the instability of the joint including dislocation or subluxation [10,11], as well as tendon rupture, excessive soft tissue removal, and poor tissue growth onto the prosthesis or allograft (Figure 1). Plain radiographs are often an initial step in imaging but may only show alignment abnormalities, while an MRI is useful for defining the extent of soft tissue failure or injury (Figure 1). Imaging can frequently reveal loosening of the endoprosthetic or allograft components secondary to the soft tissue failure. It should be noted that in the setting of multiple concurrent complications (here, also aseptic loosening), the problem primarily driving the immediate need for revision surgery defines the Henderson failure mode.

Type 1B failures are failures of coverage occurring after aseptic wound dehiscence [5,6]. These failures can occur due to factors such as excessive tension on the wound, poor wound closure, compromised blood supply to the region of the endoprosthesis or allograft, or patient factors influencing wound healing such as malnutrition and obesity. Dehiscence has also been found to occur at increased rates in the setting of perioperative radiotherapy and chemotherapy [12,13]. Patients may notice symptoms such as the edges of the wound pulling apart, increased wound drainage, and the possible exposure of underlying tissues. Despite the possibility of coverage failures leading to a deep infection, any case of inadequate soft tissue coverage leading to a deep infection and requiring revision should still be classified as type 1B [6].

The findings of wound dehiscence are clinically apparent, although cross-sectional imaging can aid in delineating the extent of soft tissue breakdown. In cases of aseptic wound dehiscence over endoprostheses, an ultrasound is well suited to identify the size and extent of an underlying fluid collection [14]. Ultimately, contrast-enhanced CT or MRI may be needed to better define deeper tissue involvement and construct integrity, with metal artifact reduction techniques employed as needed [15].

The type and incidence of soft tissue failures vary greatly as per the anatomic location of the reconstruction. For example, the most common soft tissue complication about the proximal humerus is instability, often due to the impaired strength of the surrounding muscles, which can lead to a 4% to 12% revision rate and comprise 25% to 50% of all revisions performed [5,16,17]. Similarly, instability is a common complication in proximal femoral arthroplasty due to the loss of soft tissue and muscular attachment and has been observed to occur in approximately 4% of all cases [18]. However, soft tissue failures in more distal anatomic sites, namely the distal femur and proximal tibia, are more likely to be due to a mix of instability and extensor mechanism disruption. Pala et al. found that soft tissue failures requiring revision occurred in 7% of distal femur implants and 13.3% of proximal tibia implants; while many of the distal femur failures were due to wound dehiscence, the proximal tibia failures were due to similar rates of dehiscence and extensor mechanism insufficiency [19]. Broader systematic studies have placed the rates of soft tissue failures for distal femoral reconstructions and proximal tibial reconstructions at 8.9% and 5.1%, respectively, highlighting the need for further study into the rates and nature of these failures [20].

### 2.2. Type 2 Failures—Loosening and Nonunion

#### 2.2.1. Endoprosthetic Aseptic Loosening

Type 2 failures after endoprosthetic reconstruction involve aseptic loosening [5,6]. They are further divided based on their timing after implantation, with early (type 2A) failures occurring less than two years after implantation and late (type 2B) failures occurring greater than two years after implantation. The gradual separation of the implant from surrounding bone can occur in the short term due to improper implant placement (undersizing, poor cementing technique) or adjuvant chemotherapy and radiation impeding bone ingrowth [22,23,24], or over several years due to the implant corrosion or degradation and osteoclast-mediated peri-prosthetic bone resorption [25].

**Figure 2:**
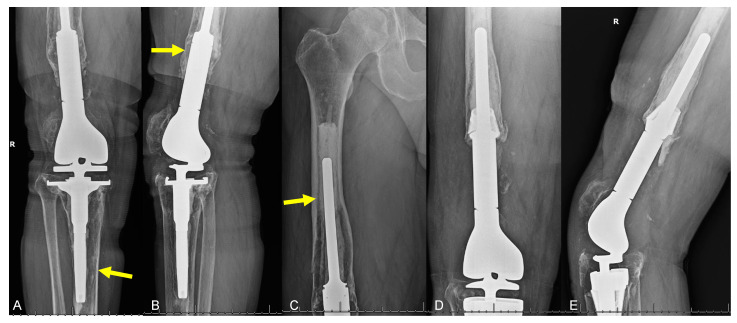
Imaging of 64-year-old male diagnosed with chondrosarcoma of the distal femur treated with resection and endoprosthetic reconstruction, presenting 13 years after reconstruction with knee pain. (**A**) AP and (**B**) lateral radiographs show aseptic loosening (type 2 failure; yellow arrows), with (**C**) extension to the femoral stem. (**D**) AP and (**E**) lateral radiographs taken post-revision with metaphyseal cones and impaction bone grafting reveal well-seated endoprosthesis without significant peri-prosthetic lucencies.

The main radiographic finding of aseptic loosening is radiolucent zones or lines at the bone–metal or bone–cement interface [1,26,27,28], as seen in Figure 2. This is in contrast to the radiographic appearance of normal and stable osseointegration, which will demonstrate an absence of lucencies at the bone–prosthesis interface and, occasionally, mild bone hypertrophy at the interface in response to compressive loading [29].

First outlined by Gruen et al., there are three main radiographic grades of aseptic loosening—possibly loose, probably loose, and definitely loose—based on the extent of the lucent lines and the level of migration of the implant, which can help determine the severity of aseptic loosening [1,26,27,28]. The definitions of the three grades are as follows:

- Possibly loose involves radiolucent zones at greater than 50% but less than 100% of the cement–bone interface;
- Probably loose involves a continuous lucent line around 100% of the cement mantle without evidence of migration;
- Definitely loose involves migration of the cement or the implant.

However, it is important to note that these classic radiographic features of aseptic loosening are more prevalent in late cases (type 2B) as compared to early cases [26].

Other radiographic findings of endoprosthetic aseptic loosening are varied and can include bone atrophy, bone hypertrophy, or reactive cortical thickening adjacent to the areas of lucency, bead shedding from the implant, distal bone pedestal (observed on imaging as dense bony sclerosis forming at the edge of the loosened prosthetic component, as an attempt to form new stabilizing bone around the implant base), trabecular attachment (observed as a bridge of endosteal bone or new trabeculae extending between the bone cortex and implant), and component subsidence (greater than 1 mm deviation, including sinking, settling, or downward translation, of the implant into surrounding tissue or bone) [28].

An MRI demonstrated excellent accuracy, sensitivity, and reproducibility in diagnosing the aseptic loosening in the total knee and hip arthroplasties, some findings of which can likely be extrapolated to the oncologic setting [30]. These include a fluid interface, osteolysis, the absence of a normal interface, poor osseous integration, and a bone marrow edema [30].

As a whole, cases of aseptic loosening make up approximately 35% of all endoprosthetic complications [31]. Generally, loosening most often occurs around implants in the proximal tibia and distal femur and more frequently affects young patients or patients with large resection length [31]. Numerous other treatment factors, implant or surgical characteristics, and radiographic findings may precede aseptic loosening. Notably, the findings mentioned above can be preceded by the radiographic findings of focal osteolysis, or the scalloping of the bone, adjacent to a component of the endoprosthesis [32]. In addition, in pediatric patients, aseptic loosening can be predicted by low levels of extracortical bone bridge ingrowth [33]. With certain types of implants such as Compress^®^ prosthetic fixation, aseptic loosening can be caused by inadequate bone growth into the porous spindle of the prosthesis or collapse at the bone–prosthesis interface [34], although these findings may overlap with type 3A failures, peri-prosthetic fractures. While peri-operative chemotherapy within 4 weeks of surgery may not increase the rate of aseptic loosening, it is still found to increase overall revision rate [35]. A crucial surgical factor that may protect against aseptic loosening is cement fixation [35].

Though prior work in smaller cohorts has found that less severe gradations of loosening are more common [32], future work may be able to analyze whether certain radiologic patterns are more prevalent with type 2A or 2B failures, or with other variables such as location, type of implant, and cementing technique (e.g., comparing newer generation uncemented press-fit stems to standard cementing technique). Imaging findings can also be compared in depth between type 2A and 2B endoprosthetic failures, to examine the possibility of earlier markers of failure necessitating revision before or after two years.

#### 2.2.2. Graft–Host Nonunion

Type 2 failures after allograft reconstructions involve allograft nonunion and are divided into hypertrophic (type 2A) and atrophic (type 2B) subtypes. Nonunion is defined as the lack of evidence of progressive healing between the native bone and the graft on follow-up imaging at nine months post-operatively [36]. Nonunion often arises in the setting of allograft placement after a tumor resection due to compromised vascular supply, host immune reaction, poor bone quality, and other patient-specific factors capable of leading to the impaired union between bone and graft, which can be worsened by adjuvant chemotherapy and radiation that can impair the survival of mesenchymal cells that are vital for joining of donor and host bone [37,38]. Symptoms of nonunion can include chronic pain at the region of the allograft that worsens with weight bearing and use of the limb [36].

Findings of nonunion may be evident on plain radiographs, and MRI and metal reduction CT can be used for further evaluation, especially when regions of sclerotic bone and surgical hardware are obscuring the site of nonunion [36].

**Figure 3:**
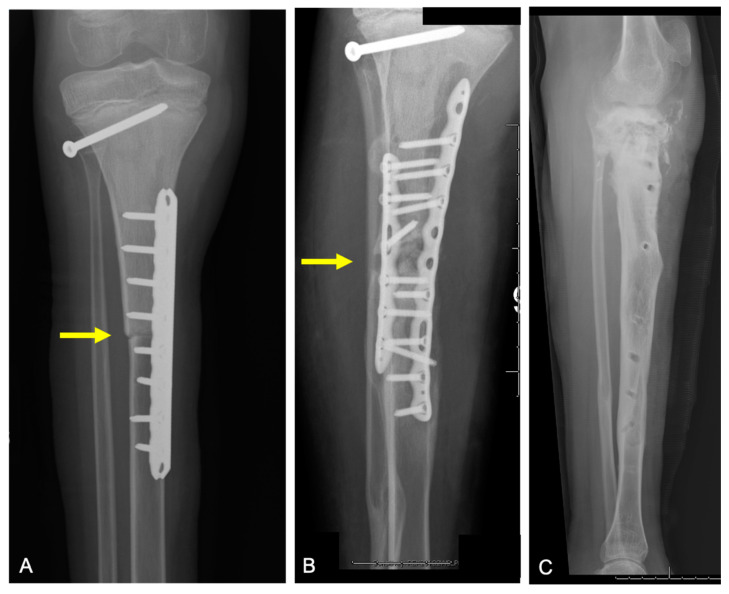
Plain radiographs in a patient with proximal tibial osteoarticular allograft placed after osteosarcoma resection, experiencing atrophic allograft nonunion (type 2B failure) and subsequent hardware loosening. (**A**) Initial radiograph 1.5 years after allograft placement demonstrates concern for nonunion with formation of fibrous tissue-filled gap between allograft and native bone (yellow arrow), and additional clinical concern for chronic infection. Allograft revision was performed, and (**B**) two-year follow-up images demonstrate persistent nonunion (yellow arrow). Subsequently, cable bone transport techniques were utilized to bridge the bone defect and achieve adequate limb length. (**C**) Radiograph after removal of all hardware (6 years after initial tumor resection) demonstrates solid regenerated bone and remodeling of the tibial shaft but a collapse of the graft articular surface.

Nonunion presents on imaging as the absence of bony trabeculae crossing the junction, persistence of lucent lines without progressive changes toward union on serial imaging studies such as radiographs, and the formation of sclerotic bone margins adjacent to the allograft (Figure 3) [36]. The major differentiating factor between type 2A and type 2B failures is the presence of abundant host-sided callus formations in hypertrophic nonunion [39]. An atrophic nonunion lacks calluses but instead shows a gap between the native bone and the allograft filled with fibrous tissue (Figure 3) [39].

The extent of bone bridging as estimated by CT carries prognostic importance—non-healing cases of nonunion typically demonstrate <5% cross-sectional bone bridging across the bone–allograft junction, whereas in cases of a healing nonunion, bone bridging is present in >25% of the cross-sectional area [37,40].

Ultimately, nonunion in allografts can occur in up to half of all cases [41,42,43,44]. Nonunion often predominantly affects femoral allograft reconstructions, as these sites experience a high degree of weight bearing and use after surgery [45]. The use of plate fixation, particularly with bridging plates rather than non-bridging plates, is associated with a lower risk of nonunion when compared to intramedullary nail-only fixation [45].

When considering other forms of biological reconstruction, namely vascularized fibular autografts, it is apparent these techniques may carry the same or slightly lower risk of nonunion. For example, Houdek et al. found that nonunion at 10 months was present in approximately 30% of patients who underwent a vascularized fibular autograft [46]. Frozen and irradiated autografts can carry a similarly lower risk of nonunion, ranging from 3% to 21% for frozen autografts [47,48] and 11.4% to 33.3% for irradiated autografts [49,50], although these rates may vary based on anatomic location.

Early detection of allograft nonunion via imaging is crucial, as up to 70% of patients can benefit from revision; however, the presence of factors such adjuvant chemotherapy, fracture, and infection can lead to further failures [37]. With further investigation of type 2 failures of allograft reconstructions, it is important to only count cases of nonunion as explicit failures when surgical intervention is necessary to facilitate union of the allograft and existing bone [51].

### 2.3. Type 3 Failures—Structural

Type 3 failures after both endoprosthetic and allograft reconstructions involve structural failure [5,6]. Patients often present clinically with significant pain and impaired movement of the limb, potentially after trauma. Structural failures are common across many types of endoprostheses, particularly megaprostheses with complex components, occurring in up to 32% of these prostheses [52].

#### 2.3.1. Structural Failure of Endoprostheses

For endoprosthetic reconstructions, structural failure can involve the breakage or wear of the implant, referred to as a type 3A failure. Type 3A failures can also encompass instances in which the lengthening mechanism of an expandable implant fails [53]. Tayara et al. found that in a series of 125 patients with cemented distal femoral endoprosthetic replacements with an all-polyethylene tibial implant for various primary and secondary tumors, 27 patients (22%) experienced type 3A failure at a mean follow-up of 7 years [54]. In a larger systematic review of revision surgeries after endoprosthetic reconstructions for extremity bone tumors, Thornley et al. stated that implant-related structural complications were found in 436 (16%) of the 2721 patients studied and constituted 33% of all failures requiring revision [7].

**Figure 4:**
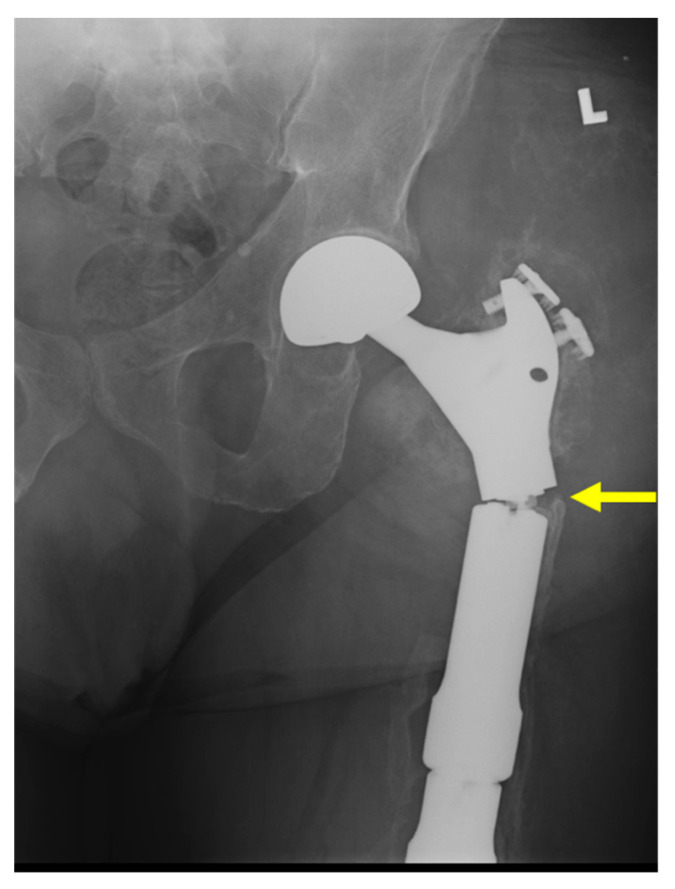
Plain radiograph of a 65-year-old male with history of femoral osteosarcoma treated with resection and endoprosthetic reconstruction, now presenting with frank prosthesis fracture (yellow arrow). Patient reported hearing a crack while standing and turning.

The radiographic appearance of endoprosthesis breakage is often clear on plain radiographs and can range from cracks along part of a device to complete prosthesis fracture (Figure 4).

**Figure 5:**
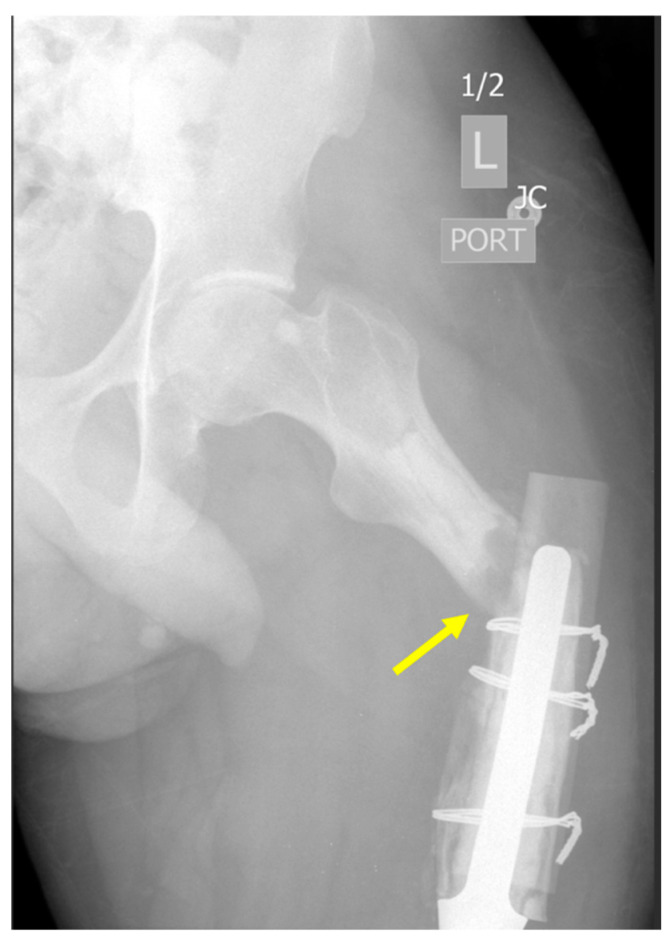
Plain radiograph of a patient with history of osteosarcoma treated with resection and endoprosthetic reconstruction, now presenting with frank periprosthetic fracture, UCS Type B2 (yellow arrow).

Type 3B failures after endoprosthetic reconstructions are structural failures at the bone, i.e., peri-prosthetic osseous fractures. Similar to type 3A failures of endoprostheses, these are widely documented in the literature and are often clearly observed on radiographs and other imaging studies, most commonly following trauma (Figure 5). Of note, there can be overlap between these type 3B failures and type 2 failures, as aseptic loosening often follows or accompanies a peri-prosthetic fracture, which may raise questions as to how some of these failures should be classified [55].

Broadly, the unified classification system (UCS) for peri-prosthetic fractures can be used for peri-prosthetic fractures related to tumor endoprostheses. This classification is based on the following radiographic features [56]:

- Type A: Fracture of an apophysis or protuberance of bone;
- Type B: Fracture involving the bed supporting or adjacent to an implant (B1, the implant is still well fixed; B2, the implant is loose; B3, the implant is loose and the bone bed is of poor quality);
- Type C: Fracture in the bone containing the implant but distant from the bed of the implant;
- Type D: Fracture affecting one bone which supports two replacements;
- Type E: Fracture involving two bones supporting one replacement;
- Type F: Fracture involving a joint surface which is not resurfaced or replaced but is directly articulating with an implant.

Importantly, this classification can help radiologists and surgeons further classify and examine peri-prosthetic fractures around tumor endoprostheses to see if certain subtypes predominate. For example, Barut et al. described 18 cases of peri-prosthetic fractures in which UCS class C peri-prosthetic fractures (“fracture which is in the bone containing the implant but distant from the bed of the implant”) were most common, totaling 67% of all fractures [57]. Further, the cumulative probability of failure (requiring a second revision) for any reason after the fracture was 27% at five years and 55% at ten years [57]. For patients treated with surgery for the initial peri-prosthetic fracture, these rates increased to 32% and 67% [57].

Aside from the UCS, more specific classifications exist to further divide peri-prosthetic fractures of a certain location or nature. For example, supracondylar peri-prosthetic femur fractures can be classified radiographically according to Su et al. as follows, where Type III fractures are most likely to require revision arthroplasty [58].

- Type I: Fracture proximal to femoral knee component;
- Type II: Fracture originating at the proximal aspect of the femoral knee component and extending proximally;
- Type III: Any part of the fracture line is distal to the upper edge of the anterior flange of the femoral knee component.

Developing these classification systems for peri-prosthetic fractures based on anatomical location has the potential to help further analyze the predisposing factors and prognosis for each fracture type.

#### 2.3.2. Structural Failure of Allografts

Structural failures have been noted to occur in up to 42% of allografts [11,41,43,44].

For allograft reconstructions, type 3A failures encompass fixation failures, in which plate or screw breakage leads to instability of the construct. These fixation failures typically arise early post-operatively before allograft–host union occurs [59], and they are usually clearly seen on plain radiographs. Of note, plate or screw breakage can and does occur in cases of nonunion, but these fixation issues should be classified as type 2 failures as they are the sequelae of long-term nonunion [43,44].

**Figure 6:**
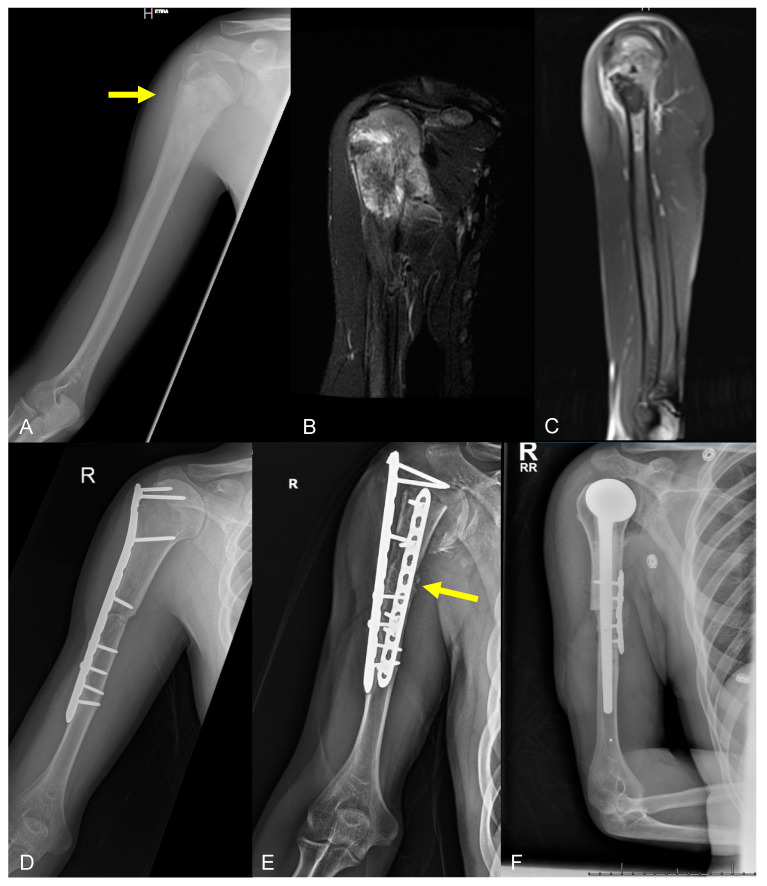
Imaging of an 18-year-old male, with history of high-grade osteosarcoma of the proximal humerus diagnosed at age 14 who received neoadjuvant chemotherapy, wide resection and osteoarticular allograft reconstruction, and revision surgery for nonunion 2 years after initial reconstruction (type 2B failure), now presenting 4 years after initial reconstruction with functional pain, found to have allograft fracture (type 3B failure). Initial (**A**) radiograph and (**B**,**C**) MR images of the tumor at diagnosis. (**D**) Radiograph demonstrating initial proximal humerus allograft reconstruction after tumor resection. (**E**) Radiograph showing allograft fracture. (**F**) Post-operative radiograph after revision to alloprosthetic hemiarthroplasty.

Type 3B failures in allografts involve fractures through the allograft, analogous to type 3A (implant breakage) fractures in endoprostheses (Figure 6). They are far more common than failures of fixation and are, once again, seen well on radiographs with further characterization through MRI and CT. With cases of allograft fractures, imaging can also help highlight specific complications such as encasement of nerves.

Similarly to cases of nonunion, cases of allograft fracture in other types of biological reconstructions occur with a lower frequency compared to traditional allografts. Fractures in cases of vascularized fibular grafts occur at rates of 3.7% to 22.2% [46,60,61,62], while those in frozen autografts can occur in 6% to 19.4% of cases [47,48] and those in irradiated autografts can occur in 6.1% to 7.4% of cases [50,63].

### 2.4. Type 4 Failures—Infection

Type 4 failures after both endoprosthetic and allograft reconstructions are due to infection [5,6]. Infection is a common complication for both types of reconstructions, and diagnosis involves a combination of clinical signs and imaging findings. Although clinical signs such as warmth, erythema, and pain are often the best indicator of infection of an endoprosthesis or allograft, they may be less evident in cases of late onset infection, making recognition via imaging of the utmost importance [64]. Infection is considered more common in allograft reconstructions as compared to endoprosthetic reconstructions, and can be a cause for failure and revision in up to 20% of proximal tibial allograft reconstructions [65]. With endoprosthetic reconstructions, there is generally a periprosthetic infection rate of 10%, with the majority occurring within 12 months of the last surgical intervention [66]. As with allografts, infections in the setting of endoprosthesis are more frequent in the lower extremities and with more distal prosthesis; they most commonly occur in the proximal tibia, followed by total femoral replacements and distal femur prostheses [64,66,67].

**Figure 7:**
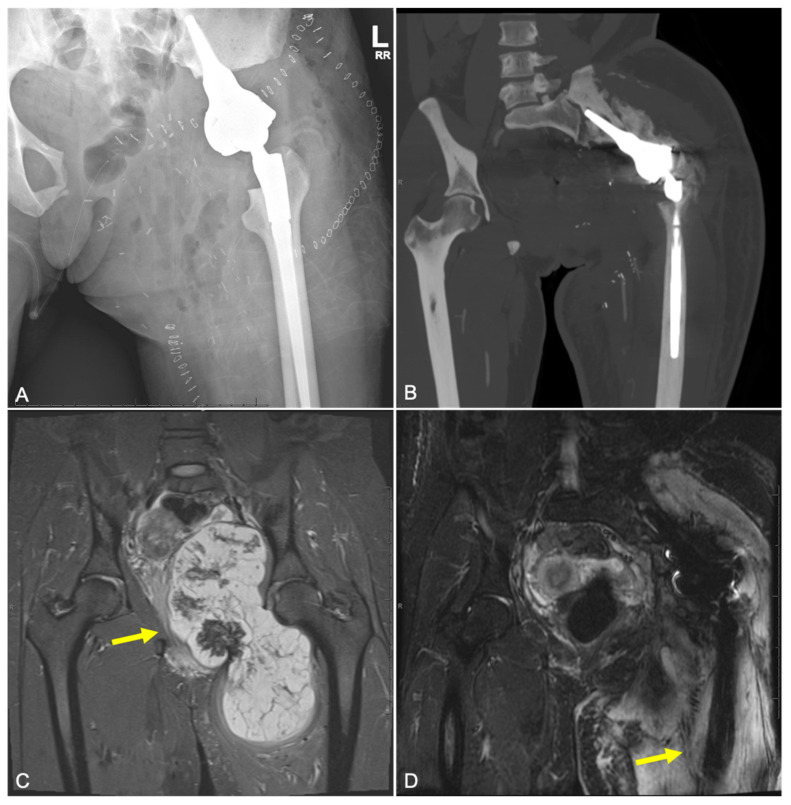
(**A**) Radiograph and (**B**) CT image of 47-year-old female patient with history of pelvic chondrosarcoma treated with internal hemipelvectomy and endoprosthetic reconstruction, now experiencing infection 2 months post-operatively (type 4A failure), requiring debridement of left hip wound. Patient previously experienced a type 1B failure, aseptic wound dehiscence, before deep infection occurred. (**C**) MR image of the original tumor (yellow arrow), measuring 20 cm in longest axis. (**D**) MR image demonstrating exposed, sclerotic left iliac bone (yellow arrow) consistent with chronic osteomyelitis as well as possible hardware loosening with fluid surrounding prosthesis stem.

The initial choice of imaging for a suspected infection is radiography, despite its low sensitivity and specificity [14,68]. They are usually used in conjunction with MRI, which is more sensitive at detecting osteomyelitis and the related soft tissue infections [14]. Although intravenous contrast is not necessary to diagnose osteomyelitis, contrast-enhanced sequences can aid in identifying and evaluating infections of the soft tissues and recognizing draining sinus tracts, fluid collections that can be drained, and tissue requiring debridement (Figure 7) [69,70]. In equivocal cases, the diagnosis of infection can be aided by CT with contrast to recognize fluid collections, effusions, and inflammation, and less commonly by FDG-PET to observe chronic infections, tagged white blood cell scan to investigate osteomyelitis, and three-phase bone scans to distinguish infection from aseptic loosening, and [14,71]. Ultrasound can aid in the evaluation and management of fluid collections, such as abscesses; in fact, joint aspiration, most commonly ultrasound guided or via fluoroscopy, is recommended alongside radiography as the initial diagnostic tool when evaluating any periprosthetic joint infections as per guidelines from the American Academy of Orthopedic Surgeons and the Musculoskeletal Infection Society [14,68,71].

Regarding endoprostheses, radiographic findings of infection are varied but can include osteolysis and lucencies at the bone–cement or bone–hardware interface greater than 2 mm [1]. Although these findings are nonspecific and can be seen in other failure subtypes, these changes are especially indicative of infection when they are rapidly progressive, irregular in nature, and accompanied with periosteal reaction or cement fractures, which can differentiate them from lucencies seen with aseptic loosening [1,72]. Further findings include complex effusions at the prosthesis sites or joints seen on radiographs, as well as complex collections that extend intra-articularly noted using MRI with metal artifact reduction techniques [8,73,74,75].

MRI findings of infection are detected with the highest sensitivity on fluid-sensitive sequences, revealing periosteal or bone marrow edema, as well as surrounding soft tissue edema and fluid collections [14]. In cases of septic arthritis, pericapsular edema and thickening on fluid-sensitive sequences is suggestive [76]. When osteomyelitis is suspected, T1 sequences are the most specific and can show confluent marrow replacement, indicated by a decreased signal intensity that will be darker than the adjacent skeletal muscle [77]. Associated cortical destruction or erosion can also be observed. Good metal artifact reduction strategies are of paramount importance in assessing the bone and soft tissues around endoprostheses [15,78].

Type 4 failures for both endoprostheses and allografts are further divided based on the timing of failure after placement of the initial implant. For endoprosthesis, type 4A failures are those occurring less than 2 years after placement, and type 4B failures are those occurring greater than 2 years after placement. For allografts, type 4A failures are those occurring less than 6 months after placement, and type 4B failures are those occurring greater than 6 months after placement.

The majority of infections cause type 4A failures, as they commonly occur close to the most recent surgical intervention, such as the primary surgery or a revision operation. For endoprostheses, a majority of the infections occur within the 2-year time range post-operatively [79]. For allografts, these infections mostly occur within the first 6 months post-operatively [80,81,82]. Any late infections (type 4B) across both endoprostheses and allografts are often in patients receiving chemotherapy and radiation [83,84].

In general, the prognosis for endoprosthesis- and allograft-associated infections leading to failure are grim; whereas mechanical complications leading to failure can often be treated successfully with revision surgery, infection is the leading cause for secondary amputation [85].

### 2.5. Type 5 Failures

Type 5 failures after both endoprosthetic and allograft reconstructions are due to local tumor recurrence or progression [5,6]. For both types of reconstructions, these failures are divided into soft tissue progression of tumor (type 5A failure) and bony progression of tumor (type 5B failure) with contamination of the implant.

Radiographs are again the most common initial imaging tool in any patient when there is concern for tumor recurrence. Imaging surveillance strategies are usually centered around radiographs and MRI, with variable use of PET-CT and ultrasound. Surveillance examinations may be as frequent as every 3 to 6 months for 2 years, semi-annually until 5 years, and annually thereafter up to 10 years post-resection, although cancer-specific protocols vary widely based on tumor histology and location, treatment history, clinical stage, and institutional practice [55,86]. Once the suspicion of recurrence is raised, biopsy confirmation may be obtained via percutaneous needle or surgical biopsy. MRI is also superior in differentiating recurrence from other phenomena that may be seen on imaging that can be confused for recurrence, including post-surgical seromas, scarring, hematomas, and inflammation [86]. FDG-PET/CT has long been considered highly sensitive for distant metastases and recurrence but may also aid in detecting local recurrence for patients with bone sarcoma despite nonspecific radiotracer uptake at treatment sites due to post-operative and post-radiation inflammation [87,88].

**Figure 8:**
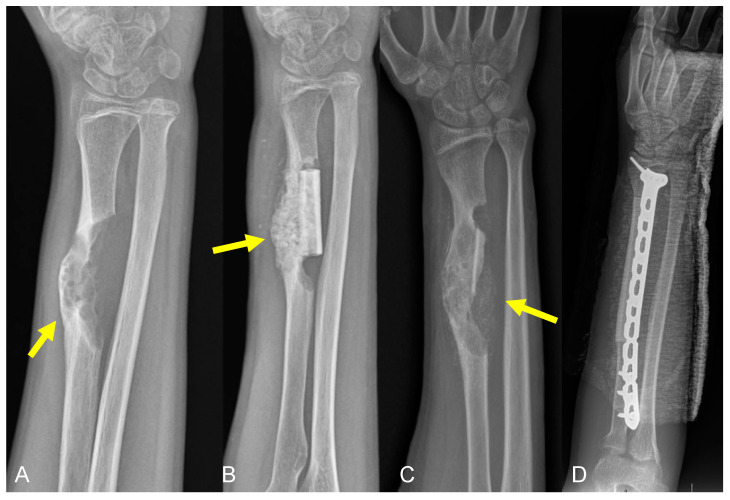
Plain radiographs of 16-year-old male with history of morphological variant of desmoplastic fibroma of the left radius (yellow arrows) treated with allograft reconstruction, now with tumor recurrence. Radiographs demonstrating (**A**) tumor recurrence after remote resection, (**B**) intralesional treatment of recurrence, and (**C**) second recurrence of tumor. (**D**) Post-operative radiograph showing repeat resection of tumor with new allograft reconstruction.

**Figure 9:**
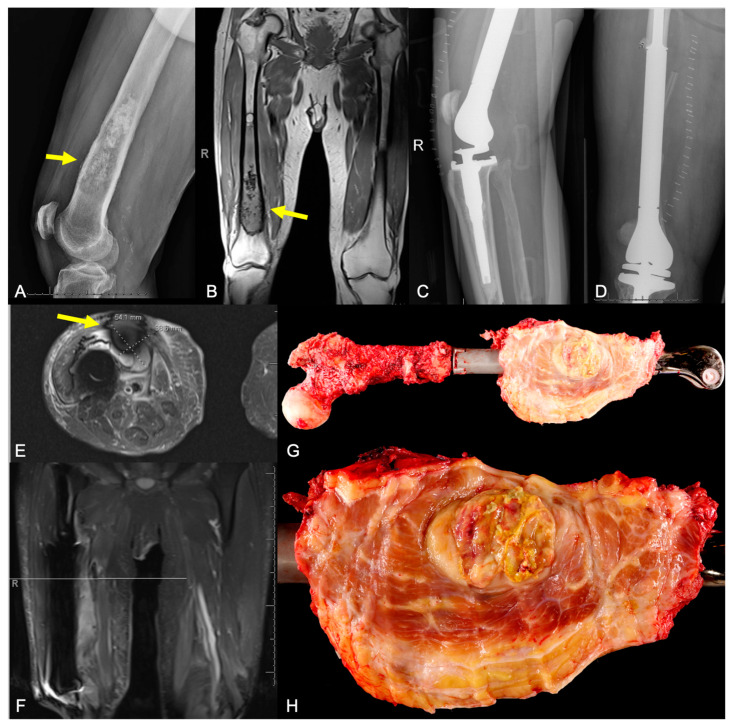
Imaging of a 77-year-old male with history of dedifferentiated chondrosarcoma of the femur treated with wide resection and endoprosthetic reconstruction, now presenting 6 months post-operatively after feeling a soft tissue mass in the region of the original tumor. Initial (**A**) radiograph and (**B**) MR image of the tumor. (**C**,**D**) Post-operative radiographs after tumor resection and endoprosthetic reconstruction. (**E**,**F**) MR images confirming tumor recurrence (type 5A failure), necessitating revision surgery, prosthesis removal, and total femoral replacement. (**G**,**H**) Gross pathology images of the removed tumor demonstrate encasement of the prosthesis.

The main imaging findings of type 5 failures are most frequently an enhancing mass in the bone or soft tissues of the surgical bed, architectural distortion if the mass is sufficiently large, and accompanying osteolysis or periosteal reaction if the recurrence involves bone (Figure 8 and Figure 9). Features of tumor recurrence are highly tumor-specific, such as the following: chondrosarcomas may exhibit little to no central enhancement because of their chondroid matrix; a similar phenomenon is observed in chordomas [89]. Osteosarcoma may recur as a calcified soft tissue nodule in or near the operative bed and be mistaken for post-operative heterotopic ossification or dystrophic calcification [90]. Myxofibrosarcoma may recur with idiosyncratic tails of infiltrative growth along fascial planes in the soft tissues [91].

Tumor recurrence in the setting of prior limb salvage surgery and reconstruction portends a poor prognosis [92]. Allograft failures due to tumor recurrence are most likely to result in both amputation and death [43,93]. Tumors are likely to recur with increased aggressiveness and often present with systemic spread [86,92]. With limb salvage surgery for osteosarcoma, studies have typically found a 7% to 10% rate of local recurrence, which is associated with close surgical margins [92]. These recurrences most commonly occur at a median of 11 to 24 months after the initial diagnosis and carry 5-year survival rates of between 13% and 40% [92,94,95,96,97,98,99]. However, while certain tumors such as osteosarcoma primarily recur locally within 2 years, others such as synovial sarcoma may recur locally after much longer periods of time, up to 15 years after diagnosis, requiring prolonged imaging surveillance [100,101].

Tumor recurrence or progression within the bone (type 5B) carries a higher risk of eventual amputation, particularly when skip metastases are present [55,102]. While cases of soft tissue recurrence can potentially be managed by wide local re-excision alongside adjuvant therapy, bone recurrence or implant contamination often requires excision of a larger portion of native bone, raising the likelihood for amputation. The decision to perform amputation is complex but key considerations are involvement of nerves and vessels, adequacy of soft tissue coverage, functionality of the prosthetic limb, overall prognosis and, ultimately, patient acceptance.

### 2.6. Type 6 Failures

Type 6 failures of limb salvage after both endoprosthetic and allograft reconstructions are pediatric failures and can be divided into physeal arrest (type 6A) or joint dysplasia (type 6B) [6]. Of note, a majority of the failures of limb salvage after tumor resection in pediatric patients fall into types 1 through 5; only specific failures in skeletally immature pediatric patients that lead to growth arrest or joint dysplasia are categorized as type 6 [103]. Revision surgeries that are performed to complete the lengthening potential of expandable prostheses are considered type 3A structural failures [6,53] and are posited to make up the majority of failures for these types of prostheses. Staals et al. described a series of 299 expandable distal femur prostheses, 102 of which had revisions for lengthening potential, comprising the most common type of failure in this sample [53]. Otherwise, there is little literature discussing the rates and imaging findings of pediatric failures.

**Figure 10:**
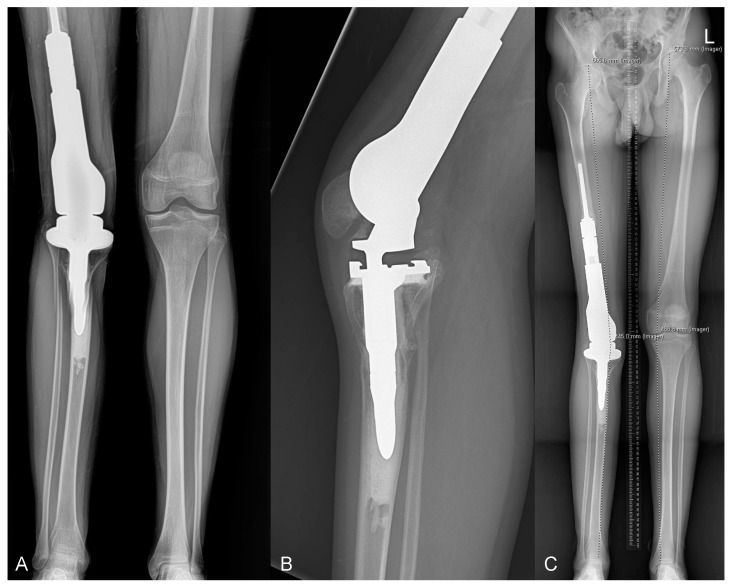
Imaging of a patient with history of high-grade osteosarcoma of the distal femur diagnosed at age 11 and treated with resection and reconstruction using Global Modular Replacement System (GMRS) extendable distal femur and hinged knee prosthesis. Patient underwent multiple standard lengthening procedures over 6 years (type 3A failures). (**A**) Leg length evaluation radiograph at age 15. Radiographs at age 16 showing (**B**) flexion contracture and arthrofibrosis and (**C**) leg-length discrepancy of 3.2 cm (type 6A failure), necessitating revision and eventual replacement with GMRS adult implant.

Type 6A failures broadly encompass any failure leading to growth arrest resulting in longitudinal or angular deformity that eventually requires revision (Figure 10). Any form of tumor resection and reconstruction surgery, particularly involving the distal femur, can cause limb shortening in children [104], and may even cause growth arrest spontaneously without a clear inciting factor [105]. In some cases, adjuvant chemotherapy may be suspected to cause a suppressive effect on bone growth [106]. Although physeal arrest can be observed clinically, radiographic findings can include unequal limbs that may lead to further deformities; for example, Zucchini et al. observed that in 23 pediatric patients with allograft reconstruction of the distal femur, 15 patients showed radiographic evidence of femoral dysmetria greater than 1.5 cm [107]. Although there is no universally accepted threshold for defining limb-length discrepancy as a longitudinal deformity, 1.5 to 2 cm is widely used as the cutoff for significant discrepancy after limb salvage. Minimal limb-length discrepancy is often expected, such as in a study of 38 patients treated with expandable endoprosthesis, Henderson et al. found that mean limb-length discrepancy was 0.7 cm [103]. Measurement of growth arrest lines, which appear as transverse radiopaque or sclerotic lines in the metaphyses of long bones on radiographs and CT images and as hypointense bands on both T1- and T2-weighted MR images, can show growth of the physis as low as 69% of that of the normal side [108].

Type 6A failures can be prevented through contralateral epiphysiodesis, to prevent further bone growth at one physis when the other has already been resected as part of endoprosthetic or allograft reconstruction.

Type 6B failures are those in which joint dysplasia occurs due to articulation with the implant. Common findings on radiographs and MRI that may suggest joint dysplasia due to the implant include evidence of joint instability such as femoral head movement, scar tissue contractures, and osteoarthritic changes of the joint [109]. To prevent hip dysplasia in the setting of expandable total femur replacements, Sevelda et al. suggested that pelvic osteotomies performed at the beginning of lengthening procedures can help avoid further hip instability [109].

Of note, a majority of the aforementioned studies do not explicitly label their failures as 6A and 6B but do mention physeal arrest or joint dysplasia. Utilizing the Henderson classification for all pediatric failures, particularly those fitting the type 6A and 6B categorization, is crucial for the further study of risk factors and outcomes after revision.

## 3. Discussions

This review has highlighted the wide range of multimodality imaging findings that delineate the various modes of reconstruction failures encountered in orthopedic oncology limb salvage surgery.

Highlighting the radiological findings associated with each failure subtype and emphasizing how they fit into a larger classification of limb-salvage complications should improve radiologists’ understanding of their surgical and prognostic implications. However, while the more common failure subtypes have clear radiologic guidelines, less common subtypes lack a clear set of comprehensive radiologic findings outlined in the literature. Adopting a uniform lexicon describing failure modes will help in identifying and prioritizing the most challenging aspects of post-operative care for limb-salvage patients, as well as drive innovation in the imaging assessment of these constructs.

We also believe it is crucial for all future work examining Henderson classification failure modes to integrate several other patient factors into analyses, including the following: Musculoskeletal Tumor Society (MSTS) functional scores, International Society of Limb Salvage (ISOLS) scores, Pediatric Outcomes Data Collection Instrument (PODCI) scores for pediatric patients, and other indicators of patient well-being, limb mobility, morbidity, and mortality after reconstructive surgery. Factoring in patient well-being is particularly important when considering pediatric patients and the high revision rates with expandable endoprostheses in skeletally immature patient; it is crucial to consider and minimize the impact of these procedures on the social and physical health of children.

Multifactorial failure that includes elements of several Henderson failure subtypes poses a challenge to unambiguous classification. In situations of overlap, for example, a case of concomitant peri-prosthetic infection and aseptic wound dehiscence (Figure 7), we emphasize that cases should be categorized into the failure mode that is the primary driver of patient symptoms and major determinant of need for revision surgery. Furthermore, there is continued discrepancy in the literature as to whether cases are counted toward a particular failure mode even if they do not result in a revision surgery, such as a patient with radiologic evidence of aseptic loosening that is not managed surgically. We favor a more restrictive definition of reconstruction failure, counting only those cases where revision surgery is performed, as this is a concrete and codable event, conducive to querying and multi-institutional database building.

Future work in this field should focus on a number of topics. One such area of focus is ensuring the Henderson classification of failure modes is as comprehensive as possible, ensuring new radiologic examples of the reconstruction cases requiring revision and new reconstructive techniques that are able to fit into a particular subtype. For example, distraction osteogenesis is a relatively novel technique for managing bone loss that shows promise in oncologic reconstructive surgery; however, while the initial results are encouraging, further long-term studies are necessary to fully evaluate its efficacy and potential failure modes [110].

Another area of future work includes analysis of the difference in failure rates and modes between the upper and lower extremities, as well as the failure rates and causes across each commonly implicated anatomic location in reconstructive surgeries, including the humerus, pelvis, femur, knee, and tibia. Specifically, much work needs to be carried out in terms of the smaller joints such as ankle and wrist, as endoprostheses are less commonly used at these sites.

In addition, as more combined resurfaced allograft–prosthetic composites are used in both pediatric and adult patients [111], the question arises as to whether failures in these implants should be classified under the categories for endoprostheses or allografts, or if a new classification should be created.

Finally, the current protocols for imaging surveillance after various endoprosthetic and allograft reconstructions are highly institutionally dependent. As more effective surveillance strategies are devised, technological improvements have the potential to better detect the development of various failure subtypes and allow for early intervention, which, in the case of failure modes such as infection, may be able to prevent revision surgery altogether.

## Figures and Tables

**Table 1:** International Society of Limb Salvage classification of failure of limb salvage after endoprosthetic reconstruction.

Category	Failure Mode	Subclassification
Mechanical	Type 1: Soft tissue failure	Functional Coverage
	Type 2: Aseptic loosening	Early Late
	Type 3: Structural failure	Implant Bone
Non-mechanical	Type 4: Infection	Early Late
	Type 5: Tumor progression	Soft tissue Bone
Pediatric	Type 6: Pediatric failures	Physeal arrest Joint dysplasia

**Table 2:** International Society of Limb Salvage classification of failure of limb salvage after biologic reconstruction.

Category	Failure Mode	Subclassification
Mechanical	Type 1: Soft tissue failure	Functional Coverage
	Type 2: Graft–host nonunion	Hypertrophic Atrophic
	Type 3: Structural failure	Fixation Graft
Non-mechanical	Type 4: Infection	Early Late
	Type 5: Tumor progression	Soft tissue Bone
Pediatric	Type 6: Pediatric failures	Physeal arrest Joint dysplasia
